# An annotated checklist of Ennominae (Lepidoptera, Geometridae) from Chiapas, Mexico

**DOI:** 10.3897/zookeys.1275.181398

**Published:** 2026-03-27

**Authors:** Andrea Murillo-Vázquez, Jorge L. León-Cortés, José Luis Rangel-Salazar, Ivonne J. Garzón-Orduña

**Affiliations:** 1 Departamento de Conservación de la Biodiversidad, El Colegio de la Frontera Sur, Unidad San Cristóbal de las Casas, Chiapas, Mexico Instituto de Biología, Universidad Nacional Autónoma de México Mexico city Mexico; 2 Laboratorio de Sistemática de Polillas, Colección Nacional de Insectos (CNIN), Instituto de Biología, Universidad Nacional Autónoma de México, Mexico city, Mexico Departamento de Conservación de la Biodiversidad, El Colegio de la Frontera Sur, Unidad San Cristóbal de las Casas Chiapas Mexico

**Keywords:** Biodiversity, Geometroidea, Mesoamerica, moths, Neotropical, phylogeny, systematics, taxonomy

## Abstract

The subfamily Ennominae represents the most diverse lineage within the family Geometridae. Knowledge about the identity and number of Mexican geometrids is limited, and particularly in regions of disproportionate Lepidoptera diversity such as Chiapas. This study provides the first comprehensive checklist of the ennomine moths from Chiapas, Mexico, a key region from the Mesoamerican biodiversity hotspot. Our checklist is derived from an exhaustive review of national and international scientific collections, digital databases, and intensive field work. A total of 239 taxa is documented: 227 named species in 101 genera, including 167 new state records and 12 unidentified taxa that likely represent undescribed entities. Representatives of 12 ennomine tribes are reported, Ennomini being the most diverse, followed by Odontoperini, Palyadini, Boarmiini, Caberini, and Macariini. The inclusion of a new combination for the genus *Besma* is discussed based on comparative genital morphology. Our results add relevant regional faunistic information and help advance the knowledge of the geometrid fauna in Mexico, and ultimately provide a key step to understanding the biogeographical origin of this diverse moth group in the Mesoamerican biodiversity hotspot.

## Introduction

For more than three decades, Lepidoptera research in Mexico has predominantly focused on members of Papilionoidea (Llorente-Bousquets 2014), leaving aside the study of highly diverse Lepidopteran groups, particularly nocturnal moths. This is noticeable in our limited knowledge on the actual diversity of the many large clades of economic and ecological importance in comparison to other North American and Mesoamerican countries (Heppner 2002; Callaghan et al. 2004; Montero-Ramírez 2010). Geometrid moths, commonly known as loopers, are the second largest family in Lepidoptera (Kristensen et al. 2007) with more than 24,000 described species. It is thought that its prominent radiation occurred in the Neotropics (Pitkin 1996; Herbulot 2001; Brehm et al. 2011), mostly driven by climatic fluctuations and more significantly by the diversification of angiosperms (Ghanavi et al. 2024).

The largest subfamily of Geometridae is the Ennominae, comprising more than 11,100 described species grouped into more than 1,100 genera. These figures account for nearly half of all geometrid species (Murillo-Ramos et al. 2019), rendering Ennominae as a highly morphologically diverse clade within the Geometridae, as well as a key lineage for understanding diversification patterns within the family and Lepidoptera as a whole. Ennomines are particularly diverse in the Neotropics at mid-elevation habitats (Holloway 1994; Brehm 2002). The evolutionary history of Ennominae (Brehm et al. 2019) reveals independent morphological and ecological shifts, particularly regarding the evolution of feeding strategies and behavioral traits, correlated with adaptations to elevational and thermal gradients (Lee et al. 2024; Ude et al. 2024). These patterns render Ennominae as a highly complex, yet poorly understood, lineage from the phylogenetic, ecological, and biogeographic perspectives.

The Mesoamerican biodiversity hotspot stands out as one of the most biodiverse areas worldwide (Myers et al. 2000), wherein the Geometridae family represents a significant component of moth diversity (Rajaei et al. 2022). Within the Mesoamerican biodiversity hotspot, Costa Rica represents a relatively well-studied region for Geometridae, with relevant advances regarding the taxonomic and ecological perspectives (Sullivan 2010, 2014; Sullivan and Chacón 2011; Matson 2023, 2024), key generic revisions (Pitkin 1993; Pitkin et al. 1996), and the recognition of elevational diversity gradients (Brehm 2007; Brehm et al. 2007; Rabl et al. 2020).

A detailed checklist of Ennominae from Costa Rica (Pitkin et al. 1996) has served as a key reference for broader revisions of Neotropical genera (Pitkin 2002). By contrast, in Mexico, research on geometrids has been scattered, mostly represented by local preliminary checklists and/or by a few generic revisions (e.g., Vázquez and Hoffman 1936; Rindge 1967, 1973; Gómez y Gómez and Beutelspacher 1999). Interest in geometrids in Mexico was revitalized in recent years, derived from systematic studies on species descriptions and natural history of native taxa (e.g., Garzón-Orduña 2021; Garzón-Orduña and Matson 2021; Garzón-Orduña and Ibarra-Vázquez 2022; Garzón-Orduña 2023, 2025; Garzón-Orduña and Brower 2025). Ideally, a well-rounded systematic study of the Mexican Geometridae should be simultaneously complemented with regional faunistic inventories like those already conducted for other Mesoamerican countries (Viidalepp and Maes 2013; Miller et al. 2012). Regional taxonomic checklists are indispensable tools for systematists because they facilitate the recognition of new species or previously unrecorded taxa, and because they are fundamental tools in guiding biodiversity assessments and developing conservation strategies.

This study provides the first systematized inventory of the Ennominae from Chiapas, the southernmost state of Mexico embedded within the Mesoamerican Neotropical hotspot. Our objective was to document the faunal composition and spatial variation of Ennominae from main vegetation associations, aiming to provide a landmark for Geometridae moth diversity and associated distributional patterns.

### The study region and its biogeographic significance in Mesoamerica

As part of the Mesoamerican biodiversity hotspot, Mexico occupies a key geographic position as a transitional zone between the Nearctic and Neotropical biogeographic regions (Morrone 2020). Studies of Mexican biodiversity have consistently documented Veracruz, Oaxaca, and Chiapas among the states with the highest species richness (SEMARNAT 2014; Falcón-Brindis et al. 2021; Cruz-Elizalde et al. 2022). Chiapas is characterized by a complex physiography that supports high biological diversity (Morrone 2005), as well as high levels of endemism of flora and fauna, particularly in mountainous areas where biogeographical barriers often limit the distribution of taxa (Liebherr 1994; Sosa and De Nova 2012; Morón et al. 2014; Dolson et al. 2024). This heterogeneity is reflected in the states’ biogeographic history, composed of lowland provinces of neotropical origin and highland provinces that are part of the Mexican Transition Zone (Morrone 2020). Its principal mountain systems, the Sierra Madre de Chiapas and the Central Highlands, are separated by the Central Depression (Müllerried 1957). Nonetheless, both ranges exhibit strong biotic affinities with the Central American Cordillera (Halffter 1987; Morrone and Márquez 2008). This regional connectivity has allowed faunal exchange between the montane forests of Costa Rica and the highlands of Chiapas, as is the case for beetles (Zunino and Halffter 1988; Beza‐Beza et al. 2021), rodents (Contreras-Medina et al. 2024), and birds (Prieto‐Torres et al. 2019; Rocha‐Méndez et al. 2024). In addition, several Neotropical Lepidoptera species extend their geographic distribution from Central America into southern Mexico, reaching transitional zones at both the Nearctic and Neotropical boundaries (e.g., Ruiz-Utrilla et al. 2018; Montañez-Reyna et al. 2023; Phelps et al. 2023). The biogeography and the natural history of geometrid moths in Mexico remains incomplete due to a shortage of specialists and lack of inventories, which deepens significant gaps of knowledge in Neotropical regions with high endemicity, and where accelerated habitat loss could lead to the extinction of yet undescribed taxa (León‐Cortés et al. 2003; Turnhout and Purvis 2020).

## Materials and methods

### Abbreviations

**BM** Bohart Museum, University of California, Davis

**CNIN** Colección Nacional de Insectos de México

**ECOSUR** El Colegio de la Frontera Sur

**ECO-SC–E** Entomological Collection of ECOSUR-San Cristóbal de las Casas, Chiapas

**GBIF** Global Biodiversity Information Facility

**MCF** Montane Cloud Forest

**NMCR** National Museum of Costa Rica

**TDF** Tropical Deciduous Forest

**TEF** Tropical Evergreen Forest

The species checklist was compiled by integrating data from four main sources: 1) verified records from global biodiversity databases (BOLDsystems.org, GBIF.org, iNaturalist.org, Fieldguide.ai); 2) a review of specialized taxonomic literature covering Neotropical Ennominae (Poole 1987; Rindge 1954, 1964a 1964b, 1967, 1972, 1973, 1978, 1990); 3) the examination of specimens at key national and international entomological collections (see above); and 4) intensive field work at three main vegetation types in Chiapas.

### Taxonomic identification

Species identifications were confirmed by comparative morphological examinations of external and internal (i.e., genitalic) diagnostic characters. External features included antennal structure, wing pattern, shape, and color. Male and female genitalia were dissected by immersing abdomens in 10% KOH and incubating it at 60 °C for 30 minutes. The abdomen was examined and dissected under a stereomicroscope OLYMPUS SZ61 and preserved in glycerin for further reference.

The taxonomy and nomenclature followed Pitkin (2002) and Brehm et al. (2019). Identifications were supported by reference specimens from the NMCR (Natural History Department, formerly InBio, Costa Rica), BM, CNIN, and ECOSUR-SC, also by consulting Scoble and Krüger (2002), Sullivan (2014), Garzón-Orduña (2023) and by checking digitized Neotropical type material from the Smithsonian National Museum of Natural History. DNA barcodes were not generated for the examined material in this study; however, reference specimens deposited at the NMCR included DNA barcode records available at BOLDsystems.org.

### Moth sampling in Chiapas

**Figure 1.**
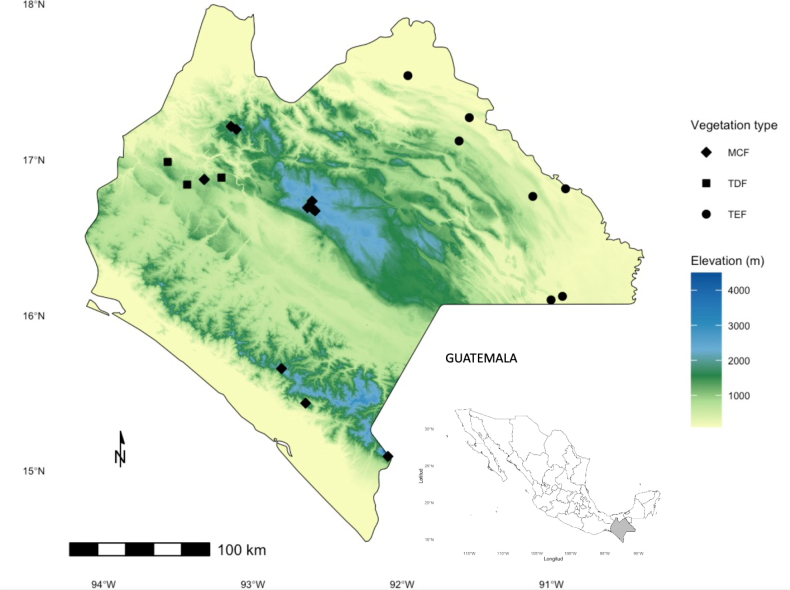
Sampling sites of Ennominae in Chiapas, México, across three vegetation types: Mountain Cloud Forest (MCF), Tropical Dry Forest (TDF), and Tropical Evergreen Forest (TEF). Background colors represent the elevation (0–>4,000 m a.s.l.).

**Figure 2.**
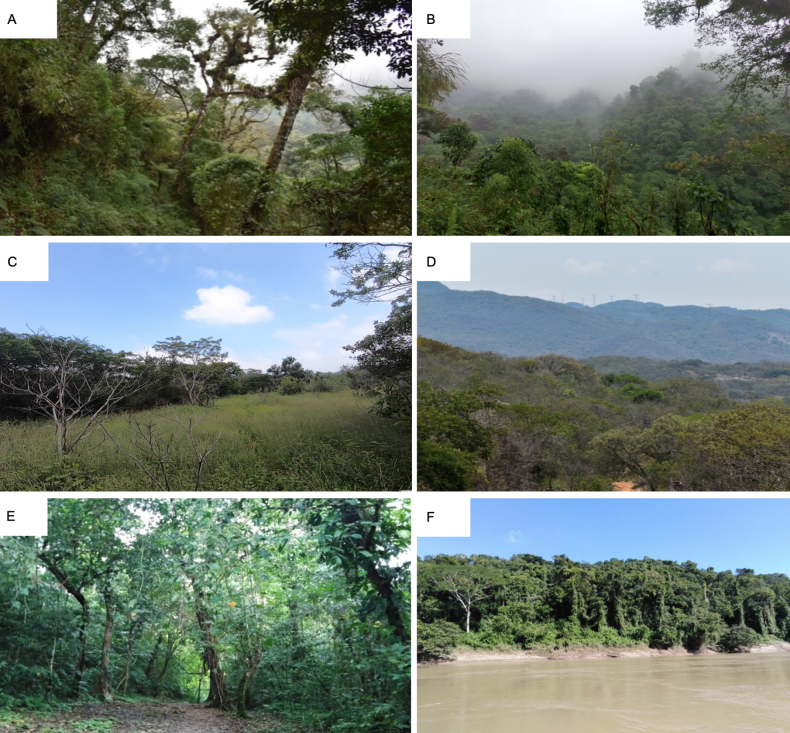
Predominant vegetation at Ennominae collection sites. **A, B**. Montane Cloud Forest on the slopes of the Tacaná Volcano; **C, D**. Tropical Deciduous Forest within the buffer zone of the El Ocote Biosphere Reserve; **E, F**. Tropical Evergreen Forest in the Lacandona Jungle, bordered by the Usumacinta River.

We collected Ennominae moths at three major vegetation types occurring across Chiapas (Rzedowski 1978). The sampling sites (Fig. 1, Table 1) span distinct physiographic provinces as recognized by Müllerried (1957). The Central Highlands (MCF): this temperate-humid region is dominated by cloud forest vegetation, including oak (*Quercus* spp. L.), fir (*Abies* spp. Mill), and pine (*Pinus* spp. L.). Sierra Madre de Chiapas (MCF) is characterized by dense evergreen vegetation and annual precipitation ranging from 2,000–5,000 mm, dominated by tropical evergreen forest, coniferous forest, and cloud forest—the latter representing one of the most important remnants of this forest type in the Mesoamerican biodiversity hotspot (González-Espinosa et al. 2011) (Fig. 2A, B). Central Depression (TDF) situated within the Grijalva River basin; this region experiences marked seasonality between dry and rainy periods, with annual precipitation ranging from 600–1,000 mm. Deciduous tropical elements dominate, particularly species from families Burseraceae, Fabaceae, and Malvaceae (Fig. 2C, D). Eastern Highlands (TEF): these are connected to the Central Highlands and span elevations from 60–1,200 m a.s.l. The region is warm-humid with predominantly tropical evergreen forest well represented in the Lacandona rainforest, including remnants of montane cloud forest (Fig. 2E, F). A geographic map for sampling sites was generated using R Studio software v. 2023.03.

**Table 1.** Ennominae collection sites in Chiapas showing predominant forest type at each location. (*) Ennominae collections made in previous years, deposited in ECO-SC-E. Elevation in meters above sea level.

Type of vegetation	Municipality	Locality	Latitude / Longitude	Elevation (approx.)
TEF	Palenque	Palenque*	17° 32.272'N, 91° 57.736'W	60
		Arroyo Xupá*	17° 16.087'N, 91° 33.051'W	90
		Frontera Corozal*	16° 48.683'N, 90° 54.371'W	110
	Ocosingo	Chajul*	16° 7.245'N, 90° 55.599'W	150
		Loma Bonita*	16° 5.852'N, 91° 0.199'W	168
		Lacanjá-Chansayab*	16° 45.747'N, 91° 7.521'W	320
		Metzabok*	17° 7.101'N, 91° 37.159'W	540
TDF	San Fernando	Vicente Guerrero*	16° 52.970'N, 93°12.691'W	900
	Ocozocoautla	Finca Corinto	16° 50.333'N, 93° 26.467'W	850
		Nueva providencia*	16° 56.142'N, 93° 38.831'W	750
MCF	Acacoyagua	Las Golondrinas*	15° 26.086'N, 92° 38.851'W	970
	Berriozábal	Trepatroncos/ La Pera	16° 52.260'N, 93° 19.620'W	1,080
	Unión Juárez	Chiquihuite	15° 5.571'N, 92° 5.747'W	1,830
	Tapalapa	Tapalapa*	17° 11.532'N, 93° 6.698'W	1,950
	Angel Albino Corzo	Reserva de la Biosfera El Triunfo (Campamento)*	15° 39.404'N, 92° 48.529'W	1,980
	San Cristóbal de las Casas	Parque Natural El Encuentro	16° 43.925'N, 92° 36.231'W	2,170
		Rancho Nuevo*	16° 40.176'N, 92° 35.030'W	2,290
		Peña Xulem	16° 41.403'N, 92° 38.112'W	2,420
		Parque Montetik	16° 40.862'N, 92° 35.952'W	2,440

Fieldwork was generally scheduled around the new moon; light traps were set along forest edges and included a mercury vapor bulb and a LepiLED UV lamp (Brehm 2017). Moths were collected manually, sacrificed in a killing jar containing ethyl acetate; sacrificed specimens were pinned and labelled following standard entomological protocols.

The completeness of the Ennominae sampling was estimated for each vegetation type and for the entire Chiapas region based on the coverage of the sample, using the iNEXT package (Chao et al. 2016). Since different vegetation associations had unequal sampling effort, we estimated sample coverage values for the observed moth diversity of each vegetation type based on the number of sampling units (incidences). In addition, we estimated sample coverage values for the observed Ennominae diversity for the entire Chiapas region based on the total number of records (individuals). In both cases, vegetation types and the Chiapas region, we estimated the number of effective species (Chao et al. 2014, Hsieh et al. 2016), where ‘0D’ indicates that values are equivalent to species richness.

## Results and discussion

A total of 239 taxa was recorded, comprising 227 species in 101 genera, including 167 new records and 12 taxa that might represent undescribed lineages (Suppl. material 1). Of these, eight correspond to known genera, but represent new species to science. The remaining four, whose suprageneric position could not be determined, were designated as incertae sedis: three were left as Ennominae spp. and one as Ennomini sp., suggesting that they could represent genera not yet described. On the other hand, of the 239 taxa, seven species were provisionally assigned to a genus and are presented with the generic name in quotation marks.

**Figure 3.**
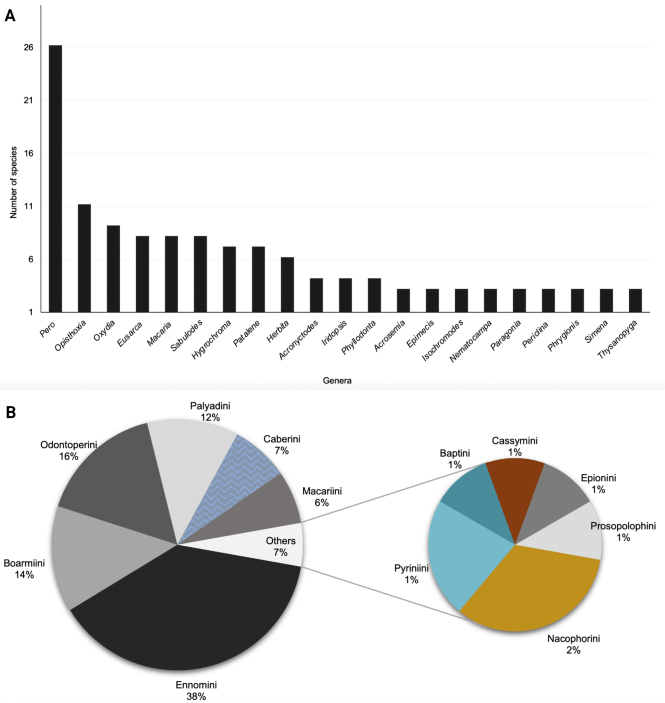
Species richness of the Ennominae fauna in Chiapas. **A**. Genera with the largest number of species (genera with only one species are not represented in the graph); **B**. Tribes with the highest representation in Chiapas (monochromatic circle).

The genera with the highest number of species are *Pero* Herrich-Schäffer (26 spp.), *Opisthoxia* Hübner (11 spp.), *Oxydia* Guenée (9 spp.), *Eusarca* Hübner, *Macaria* Curtis, *Sabulodes* Guenée (8 spp. each), *Hygrochroma* Herrich-Schäffer and *Patalene* Herrich-Schäffer (7 spp. each) (Fig. 3A). These genera were found across all sampled vegetation types, along with *Eusarca asteria* Druce, *Hygrochroma nondina* Druce, *Melanchroia chephise* (Stoll), *Nepheloleuca politia politia* (Cramer), *Opisthoxia miletia* (*Druce*), *Oxydia vesulia* (Cramer), *Patalene asychisaria* (Walker), *Phrygionis privignaria* (Guenée), *Sphacelodes vulneraria* Hübner and *Sericoptera mahometaria* Herrich-Shäffer. Among the 12 Ennominae tribes present in Chiapas, those with the highest richness were Ennomini (62 spp.), Odontoperini (26 spp.), Boarmiini (22 spp.), Palyadini (19 spp.), Caberini (12 spp.) and Macariini (11 spp.). The tribes with the lowest number of species were Nacophorini (3 spp.), Pyriniini (2 spp.), Baptini, Cassymini, Epionini and Prosopolophini (1 sp. each) (Fig. 3B).

**Figure 4.**
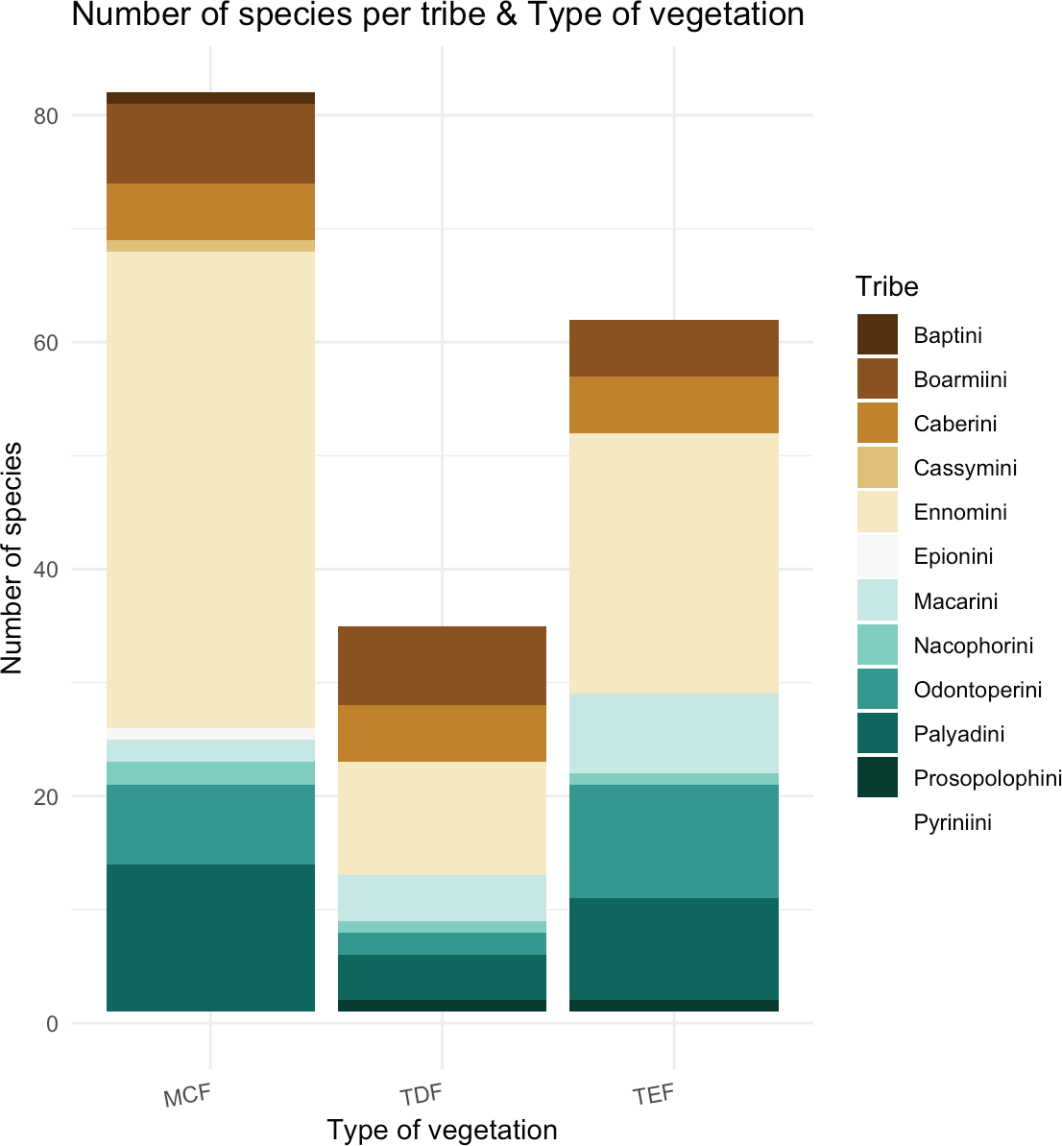
Number of species per tribe and their association with different vegetation types in the Chiapas region.

The presence of Ennominae tribes showed distinct patterns among the study vegetation types (Fig. 4). In the MCF, species of Ennomini (42 spp.) and Palyadini (13 spp.) predominated; TEF were dominated by Ennomini (24 spp.) Palyadini (10 spp.) and Odontoperini (10 spp.); and TDF displayed a more balanced representation of Ennomini (10 spp.) and Boarmiini (5 spp.). Ennomini was the only tribe with the highest representation of all three vegetation types.

**Figure 5.**
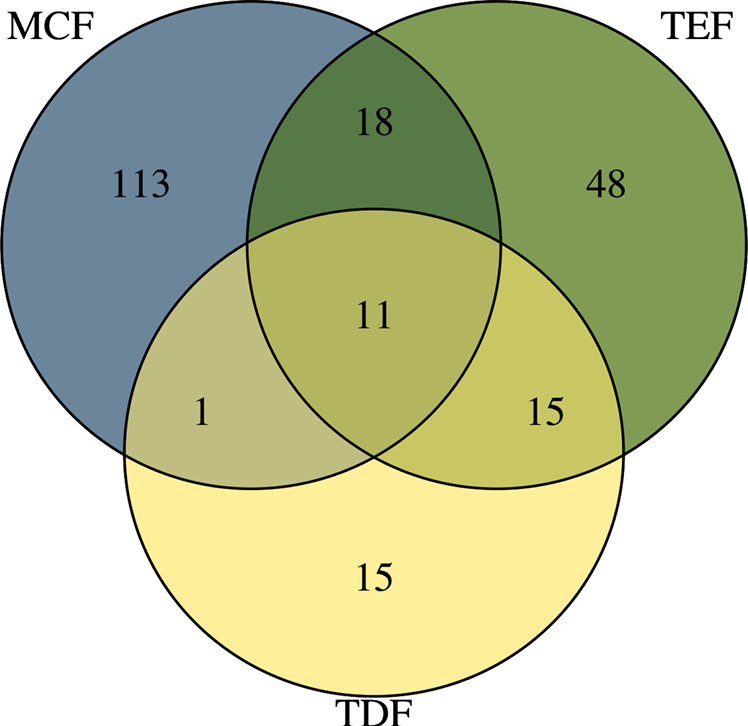
Venn diagram indicating the number of species present in each vegetation type.

**Figure 6.**
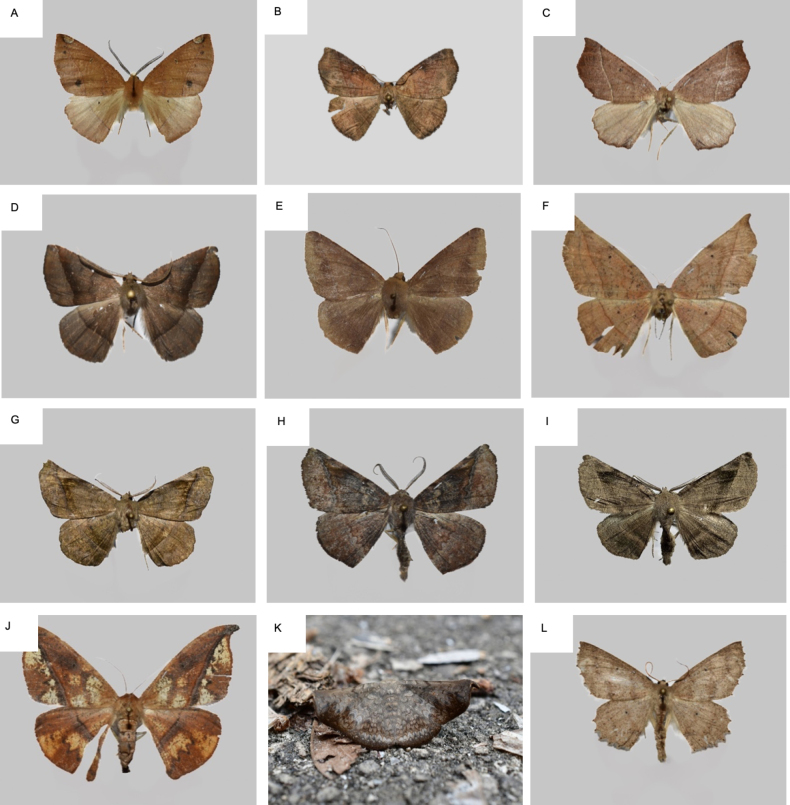
Probably undescribed Ennominae taxa from Chiapas. Photographs taken by AM-V, unless otherwise indicated. **A**. cf. *Caripeta* sp. 1 (38 mm); **B**. *Ennomini* sp. 1 (25 mm); **C**. *Ennominae* sp. 1 (38 mm); **D**. *Ennominae* sp. 2 (39 mm); **E**. *Ennominae* sp. 3 (40 mm); **F**. *Hygrochroma* sp. 1 (44 mm); **G**. *Hygrochroma* sp. 2 (32 mm); **H**. *Hygrochroma* sp. 3 (41 mm); **I**. *Hygrochroma* sp. 4 (32 mm); **J**. *Oxydia* sp. 1 (48 mm); **K**. *Patalene* sp. 1 Copyright CCBY-NC iNaturalist user coletoobservador https://mexico.inaturalist.org/observations/23378120420; L. *Isochromodes* sp. 1 (39 mm).

Faunal composition varied markedly across vegetation types. The highest number of species was recorded in high-elevation ecosystems, particularly in MCF, with 143 species, 12 of which represent undescribed taxa and 113 species are exclusive to this type of forest. In TEF, of the 92 species recorded, 48 are exclusive to tropical evergreen forests. TDF recorded 42 species, 15 of which are exclusively associated with this type of forest. The studied ecosystems sharing the most species were MCF and TEF (18 spp.), followed by TEF and TDF (15 spp.). The lowest species overlap was between MCF and TDF (1 spp.) (Fig. 5). Taxa that are probably undescribed species are shown in Fig. 6. Some species extracted from taxonomic literature (see Table 2), were not included in the vegetation type analyses or related figures, due to locality information being often ambiguous or lacking accurate collection data.

**Table 2.** Checklist of Ennominae from Chiapas. Tribal arrangement follows Brehm et al. (2019). Abbreviations: inc. sed.= incertae sedis, comb. nov = new combination, MCF = Mountain Cloud Forest, TDF = Tropical Deciduous Forest, TEF = Tropical Evergreen Forest, NA = Precise location not available. Detailed locality records for each species are included in the Suppl. material 1.

Taxa	Vegetation Type		Notes
**Tribe Baptini**			
*Lomographa candida* (Schaus, 1911)	MCF		New Chiapas Record
**Tribe Boarmiini**			
*Anavinemina indistincta* (Warren, 1906)	NA		Reported by Rindge 1964
*Epimecis conjugaria* (Guenée, [1858])	TEF		New Chiapas Record
*Epimecis puellaria* (Guenée, [1858])	MCF		iNaturalist
*Epimecis subroraria* (Walker, 1860)	MCF		New Chiapas Record
*Glena agria* Rindge, 1967	TDF		Reported by Rindge 1967
*Glena effusa* Rindge, 1967	NA		Reported by Rindge 1967
*Hymenomima camerata* Warren, 1900	TEF		New Chiapas Record
*Hymenomima memor* (Warren, 1906)	TEF, TDF		New Chiapas Record
*Hymenomima umbelaria* (Hübner, [1825])	TDF		New Chiapas Record
*Hypomecis laeca* (Schaus, 1912)	TEF, MCF		New Chiapas Record
*Iridopsis aglauros* (Schaus, 1912)	MCF		New Chiapas Record
*Iridopsis validaria* (Guenée, [1858])	TEF		New Chiapas Record
*Iridopsis herse* (Schaus, 1912)	TEF, TDF		New Chiapas Record
*Iridopsis lurida* (Schaus, 1913)	TEF		New Chiapas Record
*Melanchroia chephise* (Stoll, 1782)	TDF, TEF, MCF		New Chiapas Record
*Melanolophia bostar* (Druce, 1892)	MCF		Gómez y Gómez and Beutelspacher 1999
*Mericisca gracea* Hulst, 1896	MCF		Reported by Rindge 1972
*Nesalcis haematosticta* Dyar, 1925	TDF		New Chiapas Record
*Paraphoides largifica* Rindge, 1964	NA		Reported by Rindge 1964
*Physocleora vacillaria* (Guenée, [1858])	TDF, MCF		New Chiapas Record
Puebla aztecaria (Schaus, 1897)	NA		Reported by Rindge 1972
*Tornos brutus* Rindge, 1954	TEF, MCF		Reported by Rindge 1954
**Tribe Caberini**			
*Aplogompha argentilinea* Schaus, 1911	TEF		New Chiapas Record
*Aplogompha chotaria* Schaus, 1898	TEF		New Chiapas Record
*Lobopola cimarrona* (Dognin, 1895)	MCF		New Chiapas Record
*Parilexia nicetaria* (Guenée, [1858])	MCF		New Chiapas Record
*Parilexia proditata* (Walker, 1861)	TDF		New Chiapas Record
*Perissopteryx gamezi* Kruger & Scoble, 1992	TDF		New Chiapas Record
*Perissopteryx griseobarbipes* Kruger & Scoble, 1992	MCF		New Chiapas Record
*Sphacelodes quadrilineata* (Warren, 1900)	TDF, TEF		New Chiapas Record
*Sphacelodes vulneraria* (Hübner, [1823])	TDF, TEF, MCF		Gómez y Gómez and Beutelspacher 1999
*Thysanopyga abdominaria* (Guenée, [1858])	TDF		New Chiapas Record
*Thysanopyga casperia* (Druce, 1893)	TEF		New Chiapas Record
*“Thysanopyga” fractimacula* (Warren, 1908)	MCF		New Chiapas Record
**Tribe Cassymini**			
*Leuciris fimbraria* (Stoll, 1781)	MCF		iNaturalist
**Tribe Ennomini**			
*“Acrotomia” mucia* Druce, 1892	TEF		iNaturalist
*“Anisoperas” tessellata* (Walker, [1863])	MCF		iNaturalist
*Acronyctodes bisbili* Murillo-Vázquez, 2025	MCF		New Chiapas Record
*Acronyctodes cautama* Schaus, 1901	MCF		New Chiapas Record
*Acronyctodes colorata* (Warren, 1901)	MCF		New Chiapas Record
*Acronyctodes corrugata* Matson & Garzón-Orduña, 2025	MCF		New Chiapas Record
*Acrosemia tigrata* Schaus, 1901	TDF		New Chiapas Record
*Acrosemia undilinea* Warren, 1897	MCF		Gómez y Gómez and Beutelspacher 1999
*Acrosemia vulpecularia* Herrich-Schäffer, [1855]	MCF		New Chiapas Record
*Anisoperas lurida* Druce, 1898	TDF, TEF, MCF		New Chiapas Record
*Antepione thisoaria* Guenée, [1858]	MCF		iNaturalist
*Bassania amethystata* Walker, 1860	MCF		New Chiapas Record
*“Bassania” crocallinaria* (Oberthür, 1883)	MCF		New Chiapas Record
*Besma undularia* (Dyar, 1910) (*Endropia*)	MCF	**comb. nov**.	New Chiapas Record
*Besma mattearia* (Schaus, 1901)	MCF		New Chiapas Record
*Bonatea duciata* (Maassen, 1890)	MCF		New Chiapas Record
*Cirsodes acuminata* Guenée, [1858]	MCF		New Chiapas Record
*Destutia modica* (Schaus, 1911)	MCF		iNaturalist
Ennomini sp. 1 Duponchel, 1845	MCF	inc. sed.	New Chiapas Record
*Eusarca asteria* (Druce, 1892)	TDF, TEF, MCF		New Chiapas Record
*Eusarca crameraria* (Guenée, [1858])	TDF, TEF		New Chiapas Record
*Eusarca flexilis* (Schaus, 1912)	TDF, TEF		New Chiapas Record
*Eusarca fundaria* (Guenée, [1858])	TDF, TEF		New Chiapas Record
*Eusarca nemora nemora* (Druce, 1892)	TEF		New Chiapas Record
*Eusarca mera* (Druce, 1892)	TDF		New Chiapas Record
*Eusarca minucia* (Druce, 1892)	TEF		New Chiapas Record
*Eutomopepla artena* (Druce, 1891)	MCF		New Chiapas Record
*Eutomopepla vorda* Schaus, 1901	TEF		New Chiapas Record
*Hydatoscia ategua ategua* (Druce, 1892)	MCF		iNaturalist
*Hygrochroma* sp. 1 Herrich-Schäffer, [1855]	MCF		New Chiapas Record
*Hygrochroma* sp. 2 Herrich-Schäffer, [1855]	MCF		New Chiapas Record
*Hygrochroma* sp. 3 Herrich-Schäffer, [1855]	MCF		New Chiapas Record
*Hygrochroma* sp. 4 Herrich-Schäffer, [1855]	MCF		New Chiapas Record
*Hygrochroma bubona* Druce, 1892	MCF		New Chiapas Record
*Hygrochroma nondina* Druce, 1892	TDF, TEF, MCF		New Chiapas Record
*Hygrochroma olivinaria* Herrich-Schäffer, [1855]	TEF, MCF		Gómez y Gómez and Beutelspacher 1999
Lambdina cf. axion Druce, 1892	MCF		New Chiapas Record
*Leuculopsis intermedia* (Warren, 1906)	MCF		New Chiapas Record
*Melinodes detersaria* Herrich-Schäffer, [1855]	MCF		iNaturalist
*Microgonia perfulvata* Dognin, 1916	TEF		New Chiapas Record
*Microgonia rhodaria* Herrich-Schäffer, [1855]	TEF		New Chiapas Record
*Nematocampa arenosa* Butler, 1881	TEF		New Chiapas Record
*Nematocampa completa* Warren, 1904	TEF		New Chiapas Record
*Nematocampa reticulata* Butler, 1881	TEF		New Chiapas Record
*Neoselenia banasa* (Druce, 1892)	MCF		New Chiapas Record
*Neoselenia narcaea* (Druce, 1892)	MCF		iNaturalist
*Nepheloleuca politia iliturata* (Guenée, [1858])	TEF		New Chiapas Record
*Nephodia organa* (Druce, 1893)	MCF		New Chiapas Record
*Nephodia plautilla* (Druce, 1893)	MCF		iNaturalist
*Paragonia procidaria* (Herrich-Schaffër, 1855)	TEF		New Chiapas Record
*Paragonia cruraria* (Herrich-Schäffer, [1854])	TEF, MCF		New Chiapas Record
*Paragonia tasima tasima* (Cramer, [1779])	MCF		New Chiapas Record
Parallage nr. inconcinna Dognin, 1914	MCF		iNaturalist
*Polla hemeraria* Dyar, 1910	TDF, TEF		New Chiapas Record
*Prochoerodes tetragonata* (Guenée, [1858])	TEF		New Chiapas Record
*Sericoptera mahometaria* (Herrich-Schäffer, [1853])	TDF, TEF, MCF		Gómez y Gómez and Beutelspacher 1999
*Sicya cf. pomona* Oberthür, 1883	MCF		New Chiapas Record
*Sicya cf. inquinata* Warren, 1897	MCF		Gómez y Gómez and Beutelspacher 1999
*Simopteryx torquataria* (Walker, 1860)	TEF		iNaturalist
*Synnomos firmamentaria* Guenée, [1858]	MCF		New Chiapas Record
*Tmetomorpha bitias* (Druce, 1892)	MCF		New Chiapas Record
*Urepione quadrilineata* (Walker, [1863])	TEF		New Chiapas Record
**Tribe Epionini**			
*“Metanema” bonadea* (Druce, 1892)	MCF		New Chiapas Record
**Tribe Macariini**			
*Frederickia nigricomma* (Warren, 1904)	TDF, TEF		New Chiapas Record
*Macaria festivata* Guenée, [1858]	TEF		New Chiapas Record
*Macaria cardinea* (Druce, 1893)	TDF		New Chiapas Record
*Macaria subfulva* (Warren, 1906)	TEF		New Chiapas Record
*Macaria gambarina* (Stoll, 1781)	TEF		New Chiapas Record
*Macaria imitata* (Druce, 1893)	MCF		New Chiapas Record
*Macaria triplicaria* Herrich-Schäffer, [1855]	MCF		New Chiapas Record
*Macaria abydata* Guenée, [1858]	TDF		New Chiapas Record
*Macaria nundinata* Guenée, [1858]	TDF		New Chiapas Record
*Semiothisa divergentata* (Snellen, 1874)	TEF		iNaturalist
*Semiothisa salsa* Warren, 1905	TEF		New Chiapas Record
**Tribe Nacophorini**			
*Ischnopteris bifinita* Hübner, [1823]	MCF		New Chiapas Record
*Phaeoura spadix* Rindge, 1961	MCF		New Chiapas Record
*Thyrinteina arnobia* (Stoll, 1782)	TDF, TEF		New Chiapas Record
**Tribe Odontoperini**			
*Pero afuera* Poole, 1987	NA		Reported by Poole 1987
*Pero amanda* (Druce, 1898)	TDF, TEF		New Chiapas Record
*Pero astapa* (Druce, 1892)	NA		Reported by Poole 1987
*Pero asterodia* (Druce, 1892)	NA		Reported by Poole 1987
*Pero bulba* Poole, 1987	TEF		New Chiapas Record
*Pero caerula* Poole, 1987	MCF		New Chiapas Record
*Pero chapela* Poole, 1987	TEF		New Chiapas Record
*Pero clysiaria* (Felder & Rogenhofer, 1875)	TEF		New Chiapas Record
*Pero curuma* (Schaus, 1901)	MCF		Gómez y Gómez and Beutelspacher 1999
*Pero delauta* (Warren, 1907)	NA		Reported by Poole 1987
*Pero derecha* Poole, 1987	MCF		New Chiapas Record
*Pero dularia* Poole, 1987	TEF		New Chiapas Record
*Pero exquisita* (Thierry-Mieg, 1894)	TEF		New Chiapas Record
*Pero externa* (Warren, 1896)	TEF		New Chiapas Record
*Pero fragila* Poole, 1987	NA		Reported by Poole 1987
*Pero heralda* Poole, 1987	MCF		New Chiapas Record
*Pero lessema* (Schaus, 1901)	MCF		Gómez y Gómez and Beutelspacher 1999
*Pero melissa* (Druce, 1892)	MCF		Gómez y Gómez and Beutelspacher 1999
*Pero mnasilaria* (Oberthür, 1912)	NA		Reported by Poole 1987
*Pero nigra* (Warren, 1904)	NA		Reported by Poole 1987
*Pero plagodiata* (Warren, 1897)	TEF		New Chiapas Record
*Pero polygonaria* (Herrich-Schaffër, 1855)	TDF, TEF		New Chiapas Record
*Pero rumina* (Druce, 1892)	NA		Reported by Poole 1987
*Pero saturata* (Walker, 1867)	MCF		Gómez y Gómez and Beutelspacher 1999
*Pero registrada* Poole, 1987	TEF		New Chiapas Record
*Pero verda* Poole, 1987	MCF		Reported by Poole 1987
**Tribe Palyadini**			
*Argyrotome alba* (Druce, 1892)	TDF		New Chiapas Record
*Argyrotome mexicaria* Schaus, 1901	MCF		iNaturalist
*Opisthoxia asopis* Druce, 1892	MCF		New Chiapas Record
*Opisthoxia amabilis* (Cramer, 1777)	TEF		New Chiapas Record
*Opisthoxia bella* (Butler, 1881)	TEF		New Chiapas Record
*Opisthoxia cf. bolivari* (Oberthür, 1916)	TEF		New Chiapas Record
*Opisthoxia cassandra* Dyar, 1912	MCF		New Chiapas Record
*Opisthoxia formosante* (Cramer, [1779])	TDF, TEF		iNaturalist
*Opisthoxia miletia* (Druce, 1892)	TDF, TEF, MCF		New Chiapas Record
*Opisthoxia molpadia* (Druce, 1892)	TEF		iNaturalist
*Opisthoxia salubaea* Dyar, 1912	MCF		New Chiapas Record
*Opisthoxia uncinata* (Schaus, 1912)	MCF		New Chiapas Record
*Opisthoxia vitenaria* Schaus, 1923	MCF		New Chiapas Record
*Pantherodes unciaria* Guenée, [1858]	TEF, MCF		New Chiapas Record
*Phrygionis platinata naevia* Druce, 1892	TEF, MCF		iNaturalist
*Phrygionis polita* (Cramer, [1780])	TEF, MCF		New Chiapas Record
*Phrygionis privignaria* (Guenée, [1858])	TDF, TEF, MCF		Gómez y Gómez and Beutelspacher 1999
*Pityeja histrionaria* (Herrich-Schäffer, [1853])	MCF		Gómez y Gómez and Beutelspacher 1999
*Pityeja nazada* (Druce, 1892)	MCF		Gómez y Gómez and Beutelspacher 1999
**Tribe Prosopolophini**			
*Himeromima aulis* (Druce, 1892)	TDF, TEF		iNaturalist
**Tribe Pyriniini**			
*Acrotomia viminaria* Herrich-Schäffer, [1855]	TDF, TEF		New Chiapas Record
*Acrotomodes nr. chiriquensis* Schaus, 1901	MCF		New Chiapas Record
**Unassigned**			
*Asestra cabiria* (Druce, 1892)	MCF		New Chiapas Record
*Bagodares prosa* Druce, 1893	TEF, MCF		New Chiapas Record
*Caripeta hyperythrata* Dyar, 1910	MCF		Gómez y Gómez and Beutelspacher 1999
*Caripeta* sp. 1 Walker, [1863]	MCF		New Chiapas Record
*Cepphis megamede* (Druce, 1892)	MCF		New Chiapas Record
cf. *“Neotherinanoxiosa”* Dognin, 1917	MCF		New Chiapas Record
*Cimicodes albicosta* Dognin, 1914	TEF, MCF		New Chiapas Record
*Costalobata allidaria* (Schaus, 1901)	MCF		New Chiapas Record
*Costalobata picturata* (Schaus, 1911)	MCF		iNaturalist
*Cratoptera primularia* (Druce, 1891)	MCF		New Chiapas Record
*Cratoptera zarumata* (Oberthür, 1912)	TEF, MCF		New Chiapas Record
Ennominae sp. 1 Duponchel, 1845	MCF	inc. sed.	New Chiapas Record
Ennominae sp. 2 Duponchel, 1845	MCF	inc. sed.	New Chiapas Record
Ennominae sp. 3 Duponchel, 1845	MCF	inc. sed.	New Chiapas Record
*Erastria decrepitaria decrepitaria* (Hübner, [1823])	TEF		New Chiapas Record
*Erosina hyberniata* Guenée, [1858]	MCF		Gómez y Gómez and Beutelspacher 1999
*Euclysia angustitincta* Schaus, 1923	TEF		New Chiapas Record
*Euclysia dentifasciata* Dognin, 1910	TEF		New Chiapas Record
“*Eusarca” orsima* (Druce, 1893)	TDF		iNaturalist
*Galenara bispicula* Rindge, 1990	MCF		New Chiapas Record
*Herbita amicaria* (Schaus, 1912)	MCF		New Chiapas Record
*Herbita cyclopeata* (Möschler, 1882)	TEF		New Chiapas Record
*Herbita medona* (Druce, 1892)	MCF		Gómez y Gómez and Beutelspacher 1999
*Herbita nr. medama* Druce, 1891	TDF		Gómez y Gómez and Beutelspacher 1999
*Herbita tenebrica* Dognin, 1892	MCF		New Chiapas Record
*Herbita praeditaria* (Herrich-Schaffër, 1855)	TDF		New Chiapas Record
*Hyalostenele lutescens* (Butler, 1872)	TEF		New Chiapas Record
Ira nr. somnolenta, *1904*	MCF		New Chiapas Record
*Isochromodes cf. jodea* (Druce, 1898)	MCF		New Chiapas Record
*Isochromodes nr. granula* (Dognin, 1896)	MCF		iNaturalist
*Isochromodes* sp. 1 Warren, 1894	MCF		New Chiapas Record
*Leucula lucidaria* (Walker, 1886)	TEF, MCF		New Chiapas Record
*Leucula meganira* Druce, 1892	TEF, MCF		New Chiapas Record
*Melinodes iobarris* Dyar, 1916	MCF		Gómez y Gómez and Beutelspacher 1999
*Microxydia nr. ruficomma* Prout, 1910	MCF		iNaturalist
*Neotherina imperilla* (Dognin, 1911	MCF		New Chiapas Record
*Nepheloleuca politia politia* (Cramer, 1777)	TDF, TEF, MCF		New Chiapas Record
*Oxydia geminata geminata* Maassen, 1890	MCF		New Chiapas Record
*Oxydia gueneei* (Warren, 1904)	NA		Reported by Rindge 1957
*Oxydia masthala* Druce, 1892	MCF		Gómez y Gómez and Beutelspacher 1999
*Oxydia mexicata* Guenée, [1858]	TEF		New Chiapas Record
*Oxydia nimbata* Guenée, [1858]	NA		Reported by Rindge 1957
*Oxydia nr. obtusaria* Schaus, 1912	TEF, MCF		New Chiapas Record
*Oxydia platypterata* Guenée, [1858]	MCF		New Chiapas Record
*Oxydia* sp.1 Guenée, [1858]	MCF		New Chiapas Record
*Oxydia vesulia* (Cramer, [1779])	TDF, TEF, MCF		Gómez y Gómez and Beutelspacher 1999
*Patalene aenetusaria* (Walker, 1860)	TEF, MCF		Gómez y Gómez and Beutelspacher 1999
*Patalene asychisaria* (Walker, 1860)	TDF, TEF, MCF		New Chiapas Record
*Patalene hamulata* (Guenée, [1858])	MCF		New Chiapas Record
*Patalene* sp. 1 Herrich-Schäffer, [1854]	MCF		New Chiapas Record
*Patalene trogonaria* Herrich-Schäffer, [1856]	TEF		New Chiapas Record
*Patalene moneta* (Druce, 1892)	MCF		iNaturalist
*Patalene falcularia* (Sepp, [1852])	TEF, MCF		iNaturalist
*Periclina apricaria* (Herrich-Schaffër, [1855])	MCF		Gómez y Gómez and Beutelspacher 1999
*Periclina merana* (Schaus, 1911)	MCF		iNaturalist
*Periclina syctaria* (Walker, 1860)	MCF		New Chiapas Record
*Phyle aspilotos* Rindge, 1990	MCF		New Chiapas Record
*Phyle schausaria* (Edwards, 1884)	NA		Reported by Rindge 1990
*Phyllodonta flabellaria* Dyar, 1912	MCF		New Chiapas Record
*Phyllodonta druciata* Schaus, 1901	MCF		New Chiapas Record
*Phyllodonta indeterminata* Schaus, 1901	TEF, MCF		New Chiapas Record
*Phyllodonta esperanza* Sullivan, 2014	MCF		New Chiapas Record
*Prochoerodes pilosa* Warren, 1897	TDF, TEF		New Chiapas Record
*Rhomboptila nr. cajanuma* (Dognin, 1892)	TEF		New Chiapas Record
*Rindgeria picta* (Schaus, 1898)	MCF		New Chiapas Record
*Sabulodes cf. arge* Druce, 1891	MCF		New Chiapas Record
*Sabulodes exhonorata* Guenée, [1858]	NA		Reported by Rindge 1978
*Sabulodes nr. matrica* Druce, 1891	MCF		New Chiapas Record
*Sabulodes loba* Rindge, 1978	TEF, MCF		Reported by Rindge 1978
*Sabulodes setosa* Rindge, 1978	TEF		Reported by Rindge 1978
*Sabulodes solola* Rindge, 1978	NA		Reported by Rindge 1978
*Sabulodes striata* Rindge, 1978	NA		Reported by Rindge 1978
*Sabulodes plauta* Rindge, 1978	MCF		Reported by Rindge 1978
*“Selenia” mariaria* Schaus, 1927	MCF		New Chiapas Record
*Simena schusteri* Covell & Heppner, 2017	MCF		New Chiapas Record
*Simena umbrifera* Schaus, 1901	MCF		New Chiapas Record
*Simena luctifera* Walker, 1856	TEF, MCF		New Chiapas Record
*Trotopera arrhapa* (Druce, 1891)	TEF		New Chiapas Record

**Figure 7.**
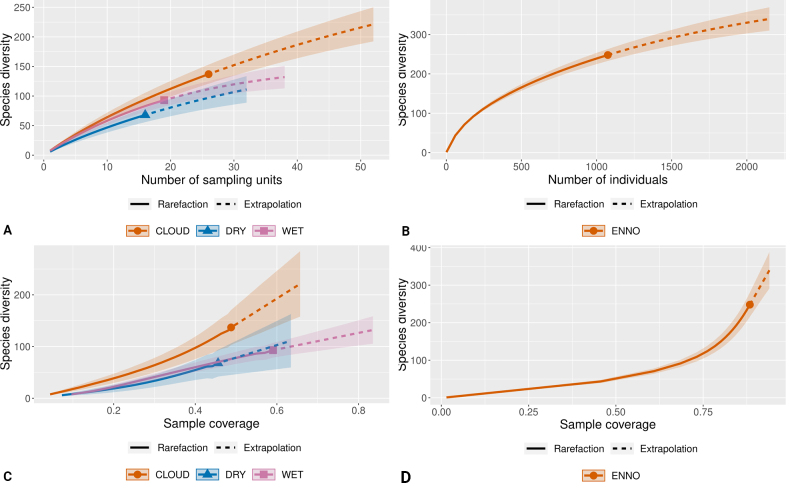
Ennominae moths’ incidence/individual-based rarefaction and extrapolation for Hill diversity indices and Sample coverage values for each vegetation type. (**A, C**); (names as in Table 1) and for the entire Chiapas (**B, D**). 0D, values are equivalent to species richness (**A, B**). Shaded regions represent 95% confidence intervals based on 200 bootstrap replicates (Chao et al. 2014).

The rarefaction and extrapolation curves revealed differences for levels of species diversity recorded among forest types. The MCF showed the highest diversity of Ennominae, followed by the TEF, while the TDF had the lowest values (Fig. 7A, C). The estimated coverage of the sample (*C. hat*) ranged from 0.46 (TDF) to 0.59 (TEF) for forest types. At the regional scale, the accumulation curve did not reach an asymptote, indicating that further sampling would likely reveal potential additional species (Fig. 7B, D). However, the coverage sample for the regional moth fauna scored 0.88. In general, we were confident to compare the cumulative moth richness and diversity among forest types and for the entire Chiapas region at comparable levels of sampling effort.

Of the 239 taxa recorded for the Chiapas state, the highest species diversity was found in montane ecosystems, particularly in MCF, with 143 species, being 113 exclusives to this forest type. This ecosystem is the most diverse and includes the highest number of endemic species. In contrast, TEF and TDF showed lower diversity, containing 92 and 42 species, respectively. The most species-rich genera in our dataset were *Pero*, *Opisthoxia*, *Oxydia*, *Eusarca*, *Macaria*, *Sabulodes*, *Hygrochroma*, and *Patalene*. These genera are found across the studied vegetation types suggesting a broad tolerance to different environments. In contrast, *Hygrochroma* and *Oxydia* showed highest species richness in montane vegetation types, indicating more specialized ecological requirements. Our checklist adds 167 new records in Chiapas, representing an important addition to the known regional fauna (i.e., Gómez y Gómez and Beutelspacher (1999) previously documented 22 Ennominae species from a single location in Chiapas); however, we abstain of doing a comparison with other localities in Mexico because the results of this study indicates that variation in sampling effort has a substantial effect on the size of a checklist (León-Cortés et al. 1998).

Previous studies reported highest levels of Ennominae diversity at mid-highland habitats i.e., 600–1,000 m a.s.l. in tropical countries such as Borneo and Ecuador (Holloway 1994; Brehm 2002). Our results indicate lower species richness at lower elevations and a notably higher proportion of endemics above 1,500 m a.s.l., with peak diversity levels occurring above 1,200 m a.s.l. This underscores the ecological importance of montane ecosystems in Chiapas as reservoirs of Geometridae diversity. Although the number of high-elevation sampling sites was comparatively greater, this was offset by intensive fieldwork conducted in the Lacandona Rainforest during the 1990s by JL L-C. Nevertheless, species richness in lowland forests remained substantially lower than in montane habitats. The relatively low species richness of ennomines recorded in TDF may also be related to the limited availability of food resources, driven by the phenology of the vegetation (van Asch and Visser 2007; Macphie et al. 2025).

### Vegetation associations and host plants recorded

Host plant preferences among Ennominae documented by Brehm (2002) and Bodner et al. (2010), suggested a marked association with woody plants, and particularly for members of the families Asteraceae, Araliaceae, Euphorbiaceae, and Myrsinaceae, with many species exhibiting polyphagous habits. *Eusarca asteria*, *Melanchroia chephise*, *Oxydia vesulia*, *Patalene asychisaria*, *Sericoptera mahometaria*, and *Sphacelodes vulneraria* were recorded across all sampled vegetation types, consistent with their wide host-plant breadth and their tolerance to environmental heterogeneity.

In addition, our results provide new records of herbaceous plants as Ennominae larval food sources: *Sicya* spp. was observed feeding on Grossulariaceae, *Costalobata allidaria* (Schaus) reared by us and fed on coffee leaves (*Coffea* sp.) and larvae of *Hygrochroma* spp., displayed generalist feeding behavior of host plants located at high elevations (*Buddleja* spp., *Coffea* spp., and *Crassocephalum* spp.). Monophagy appears less common among Ennominae members; for example, species of *Acronyctodes* Edwards have been recorded only from *Buddleja* spp., but more recent records include *Schelehelia parviflora* (Oerst) in Costa Rica and *Cymbalaria* spp., all three within the order Lamiales (Garzón-Orduña et al. 2025). These findings suggest that the distribution and availability of host plants likely shape the occurrence and success of Ennominae in Chiapas and throughout Mesoamerica (Brehm 2002), in line with has been reported for the genus *Eois* Hübner in the Neotropics (Jahner et al. 2017). Further research on the natural history of Ennominae is needed to achieve a more complete understanding of Ennominae’s ecology in Mexico.

At the tribal level, Ennomini and Palyadini were the most diverse clades, the latter being almost exclusively Neotropical except for *Phrygionis*, which has been recorded in the southern United States (Scoble 1994). Ennomini was widely distributed across tropical and montane ecosystems, while Palyadini was primarily associated with MCF. In contrast, Odontoperini were particularly prominent in TEF, and generalist species of Boarmiini, Caberini and Macariini were common in the Central Depression of Chiapas —characterized by dry, disturbed environments and TDF, where they are frequently associated with Fabaceae host plants, a pattern also noted by Brehm (2002). Although Palyadini was better represented in montane forests; this pattern could be partially influenced by sampling bias. However, the known distributions of its potential host plants in high-elevation habitats such as species of *Ardisia* and *Myrsine* L. (Myrsinaceae) (Scoble 1995; Bussmann 2001; Pipoly and Ricketson 2005; observations in iNaturalist.org) support the idea of a potential ecological affinity of this group with montane environments.

Our results indicate that the greatest species overlap occurred between MCF and TEF, probably because some collecting sites were located along forest ecotones, where vegetation gradually changes from MCF to lowland rainforest. Such transitional zones may have contributed to the apparent overrepresentation of Palyadini in montane habitats. In general, the suprageneric structure observed in Chiapas only partially aligns with the Neotropical pattern reported by Brehm et al. (2019), where Ennomini and Boarmiini dominate. Notably, quite a few genera in our study remain unassigned to a tribe, which may not accurately reflect the true diversity of the subfamily.

### Distribution patterns of Ennominae in Mesoamerica

Costa Rica shares similar biogeographic characteristics with Chiapas, but differs in having decades of taxonomic research, consolidated collections, and active taxonomic specialists (Janzen and Hallwachs 2016; Quicke et al. 2024). We revised the Ennominae checklist from Costa Rica provided by Pitkin et al. (1996) as a reference for assessing our species checklist from Chiapas. At least 597 Ennominae species have been documented in Costa Rica. By contrast, Chiapas, 1.3 times larger in area, has recorded 239 species. While this number is significant, it still reflects an underrepresentation of the actual regional species diversity levels. According to observations in GBIF.org, and records from the NMCR, several Ennominae species are shared between both regions. The complex topography of Chiapas in the Mexican Transition Zone, appears to function as a biogeographic bridge facilitating faunal exchange among closely related lineages (Ray et al. 2006; Morrone 2020). For instance, *Acronyctodes asignum* Matson and *A. corrugata* Matson & Garzón-Orduña, exhibit broad distributions along the Central American Cordillera (Garzón-Orduña et al. 2025). Similarly, species of *Phyllodonta* in the *latrata* complex inhabit a wide elevational range, from 500–2,200 m a.s.l. (Sullivan 2014; Garzón-Orduña and Brower 2025) throughout the montane system. Recent biogeographic analyses of Geometridae (Ghanavi et al. 2024) suggest a Nearctic origin for the most recent common ancestor (MRCA) of Ennominae, which may explain their affinity with temperate environments and subsequent dispersal into the Neotropics. This pattern is reflected in the distributional limits of several Ennominae species: some with Nearctic affinities -such as *Antepione thisoaria* (Guenée) and *Caripeta hyperythrata* Dyar- reaching their southernmost limit in Chiapas; whereas Neotropical species such as *Bagodares prosa* Druce, *Bonatea duciata* Maassen, *Paragonia tasima* (Cramer), *Rhomboptila cajanuma* Dognin, *Tmetomorpha bitias* Druce, and *Trotopera arrhapa* Druce, reach their northernmost distributions in Chiapas. This is also consistent with previous findings for other lepidopteran taxa reported for the Mesoamerican biodiversity hotspot (León-Cortés 2000; Moguel-Cárdenas et al. 2024).

Overall, the composition of the Ennominae fauna in both Chiapas and Costa Rica follows a pattern of biogeographic continuity across the Mesoamerican highlands (Morrone 2006; Salom-Pérez et al. 2021). Despite contrasting geological histories between Chiapas and Costa Rica, which may have produced divergent evolutionary processes within Ennominae, including allopatric speciation promoted by major physiographic barriers such as the Nicaraguan Depression and the Isthmus of Tehuantepec in Mexico (see Smith et al. 2012; Bacon et al. 2015; Jiménez and Ornelas 2016; Amador et al. 2024). Future phylogeographic studies on Ennominae could offer valuable insights into the evolutionary relationships and distributional patterns of this group across Mesoamerica.

## Conclusions

This study represents the first regional checklist of the subfamily Ennominae (Geometridae) in Chiapas, documenting 239 species in 101 genera, including 12 potentially new taxa. Of the total number of taxa reported, 167 represent new state records, prompting us that a considerably greater diversity is expected than previously known (22 species reported by Gómez y Gómez and Beutelspacher (1999) and 25 reported in previous generic revisions of Neotropical taxa). The recorded faunal composition suggests that Chiapas represents a biogeographic bridge within the Mexican Transition Zone, where taxa of Nearctic and Neotropical origin converge. That is, taxa with Nearctic affinities reach their southern distribution limit in the mountain forests of Chiapas, while those with Neotropical affinities find their northern limit in the tropical forests.

Our study provides a baseline for generating inventories in other regions and establishing comparisons at different biogeographic scales. More taxonomic work on Ennominae in the Neotropical region is definitely needed. It is hoped that these potential new taxa will be validated in the future through complementary analyses, such as barcoding, and that new sampling will continue to enrich the inventory of geometrids in the state. Furthermore, our results provide a landmark for guiding conservation strategies, particularly in montane forests, and serve as a reference for future phylogenetic studies on Ennominae in the region.

### Taxonomic notes

The following taxa are of Nearctic and Neotropical affinity with distribution ranges that extend into the mountainous areas of Chiapas. These cases represent significant expansions of their distribution into the Mexican Transition Zone.

#### *Hypomecis* Hübner, 1821

Pitkin (2002) indicated that *Hypomecis* is primarily an Old-World genus. Some species, such as *H. umbrosaria* Hübner, may represent Nearctic taxa. The placement of Neotropical species currently assigned to *Hypomecis* is likely erroneous. *Hypomecis laeca* Schaus (*Nesalcis*), recently validated by Rajaei et al. (2022), supports this reassessment.

#### Nacophorini s.l.

According to morphological studies by Pitkin (2005), the monophyly of Nacophorini is weakly supported. Brehm et al. (2019) noted that numerous species from across the globe have been historically assigned to the tribe (sensu stricto); however, their phylogenetic analysis recovered Nacophorini as a strictly New World clade. The authors therefore propose a sensu lato concept, encompassing exclusively Neotropical taxa, including genera such as *Thyrinteina* Möschler, *Phaeoura* Hulst, *Holochroa* Hulst, and *Betulodes* Thierry-Mieg. In our checklist, we adopt the sensu lato term, given the presence of *Ischnopteris* Hübner, a genus restricted to the Neotropics.

### Examined material

Taxonomic considerations are presented for taxa that could not be assigned to described species or genera within Ennominae. In addition, possible new species and a new combination are evaluated.

Specimens left as Ennominae sp. and Ennomini sp. did not match any previously described morphotypes, thus they were conservatively assigned to the subfamily and tribal level. These specimens may represent highly differentiated species, and confirming their taxonomic identity will require additional data, such as COI barcodes.

#### *Bassania* Walker, 1860

Pitkin (2002) noted that not all members of *Bassania* share the diagnostic apomorphy of the genus, specifically the pale sheen in the medial area and the oblique postmedial line on the FW. We examined the male genitalia of “*Bassania*” crocallinaria (Oberthür), a species that Pitkin excluded from *Bassania* based on morphological differences in the genital structures. She also observed a gnathos with a weak central connection, resulting in two groups of spines whose number varies among specimens. Unlike *B. amethystata*, the type species of the genus, *B. crocallinaria* lacks a furca and instead possesses a tongue-like medial extension of the juxta. Additionally, other specimens morphologically like *B. crocallinaria* and sharing comparable genital traits were examined. However, due to the uncertainty in their specific delimitation, these are provisionally referred to as Ennominae sp. 3 (Fig. 6E).

#### *Besma undularia* Dyar, 1910, comb. nov.

Pitkin proposed that *Euchlaena undularia* Dyar, (*Endropia*) a Nearctic species, might be more appropriately placed within *Besma* Capps. Our study confirms this reassignment, formally proposing the new combination *Besma undularia*. The transfer is supported by comparative morphology of male genitalia with the type species of *Besma* (which is *Besma quercivoraria* (Guenée, [1858]) (*Metanema*). Adults of *B. quercivoraria* and *E. undularia* share wing similarities: the outer margin of forewing and hindwing is angled at vein M3, and they share a characteristic brownish-orange coloration. The male genitalia of *B. undularia* are consistent with the diagnosis of genus *Besma* according to Pitkin (2002): gnathos broad, densely spinulated midsection and furca deflected to right. This contrasts with male genitalia of *Euchlaena* Hübner, whose males lack a furca, have a weak gnathos and possess valves with spine-like processes.

#### Ennomini sp. 1

Specimens from this group exhibit predominantly ochre coloration with orange tones (Fig. 6B). Their assignment to the Ennomini tribe is supported by the presence of a spinose furca deflected to the left on the male genitalia, and median plate of gnathos with a triangular shape, oriented upwards with the outer edge spinulose.

#### *Hygrochroma* Herrich-Schäffer, [1855]

Differences in the male genitalia among specimens superficially resembling *H. bubona* Druce from Chiapas suggest the presence of a cryptic species complex. While these specimens share external features such as dorsal and ventral wing pattern, the difference in the male genitalia—such as the variation in furca morphology—indicate the presence of more than one undescribed species.

#### cf. Neotherina noxiosa Dognin,1917

The examined male specimen exhibits wing maculation typical of *N. noxiosa* and bipectinate antennae. However, the male genitalia lack the characteristic right-oriented furca of *Neotherina* Dognin, instead presenting a spinose process from the juxta and a medially extended, spinulose gnathos. Pitkin (2002) excluded this species from *Neotherina* due to the absence of synapomorphies and proposed *Nephodia* Hübner as a possible alternative. Nonetheless, our specimen does not exhibit the defining genital features of *Nephodia* either.

#### “Selenia” mariaria Schaus, 1927

Although *Selenia* Hübner is documented as a genus occurring in the Old World and North America, we collected specimens of *S. mariaria*, whose type locality is Guatemala, in montane forests, raising questions about its taxonomic placement. Pitkin (2002) excluded *S. mariaria* and other neotropical species from *Selenia* and suggested they might belong to cf. *Neoselenia* Pitkin or to another genus. The male genitalia of *S. mariaria* are not consistent with diagnosis described by Pitkin for *Neoselenia*. The taxonomic circumscription of *Neoselenia* and the status of these orphan species are subject of current investigation (Garzón-Orduña in prep.)

## Additional information

### Use of AI

No use of AI was reported.

### Author ORCIDs

Andrea Murillo-Vázquez https://orcid.org/0009-0004-4222-4667

Jorge L. León-Cortés https://orcid.org/0000-0003-1706-5620

Ivonne J. Garzón-Orduña https://orcid.org/0000-0003-3914-4952

### Data availability

All of the data that support the findings of this study are available in the main text or Supplementary Information.

## Supplementary materials

Supplementary material 1 Locality data of Ennominae taxa examined and documented for Chiapas in this study Data type xlsx Explanation note The supplementary file provides the locality data as found in the labels of the ennomine taxa documented for Chiapas in this study. https://binary.pensoft.net/file/1571891This dataset is made available under the Open Database License (http://opendatacommons.org/licenses/odbl/1.0/). The Open Database License (ODbL) is a license agreement intended to allow users to freely share, modify, and use this Dataset while maintaining this same freedom for others, provided that the original source and author(s) are credited. Andrea Murillo-Vázquez, Jorge L. León-Cortés, José Luis Rangel-Salazar, Ivonne J. Garzón-Orduña
